# Sex-Related Differences in Innate and Adaptive Immune Responses to SARS-CoV-2

**DOI:** 10.3389/fimmu.2021.739757

**Published:** 2021-10-20

**Authors:** Sudhanshu Agrawal, Jon Salazar, Thu Michelle Tran, Anshu Agrawal

**Affiliations:** Division of Basic and Clinical Immunology, Department of Medicine, University of California Irvine, Irvine, CA, United States

**Keywords:** SARS-CoV-2, dendritic cells, CXCL-10, interferon-α, monocytes, sex differences

## Abstract

Coronavirus disease 2019 (COVID-19) exhibits a sex bias with males showing signs of more severe disease and hospitalizations compared with females. The mechanisms are not clear but differential immune responses, particularly the initial innate immune response, between sexes may be playing a role. The early innate immune responses to SARS-CoV-2 have not been studied because of the gap in timing between the patient becoming infected, showing symptoms, and getting the treatment. The primary objective of the present study was to compare the response of dendritic cells (DCs) and monocytes from males and females to SARS-CoV-2, 24 h after infection. To investigate this, peripheral blood mononuclear cells (PBMCs) from healthy young individuals were stimulated *in vitro* with the virus. Our results indicate that PBMCs from females upregulated the expression of HLA-DR and CD86 on pDCs and mDCs after stimulation with the virus, while the activation of these cells was not significant in males. Monocytes from females also displayed increased activation than males. In addition, females secreted significantly higher levels of IFN-α and IL-29 compared with males at 24 h. However, the situation was reversed at 1 week post stimulation and males displayed high levels of IFN-α production compared with females. Further investigations revealed that the secretion of CXCL-10, a chemokine associated with lung complications, was higher in males than females at 24 h. The PBMCs from females also displayed increased induction of CTLs. Altogether, our results suggest that decreased activation of pDCs, mDCs, and monocytes and the delayed and prolonged IFN-α secretion along with increased CXCL-10 secretion may be responsible for the increased morbidity and mortality of males to COVID-19.

## Introduction

The coronavirus disease 2019 (COVID-19) pandemic has affected millions of individuals worldwide (1, 2). The majority of infected individuals have mild illness, and many may be asymptomatic (1, 2). Those with serious illness developed severe respiratory complications associated with increased proinflammatory cytokines including CXCL-10, CCL-2, TNF-α, etc. in the plasma (3, 4). This so-called “cytokine storm” can initiate viral sepsis and inflammation-induced lung injury which lead to other complications including pneumonitis, acute respiratory distress syndrome (ARDS), respiratory failure, shock, and potentially death (3, 4). Comorbidities include age, diabetes, hypertension, heart disease, etc. (2, 5, 6). Another noticeable feature of the pandemic is the difference in morbidity and mortality observed between sexes. This has been documented by several epidemiological studies. Though the number and age of males and females with COVID-19 infection are comparable, males tend to display more severe disease (7–9). The odds of hospitalization, ICU admission, and mortality were nearly three times higher in male patients as compared with those in females (8, 10). The underlying mechanisms are unclear.

Several factors have been speculated to account for the disparity including differences in biology, behavior, occupation, etc. (7). Changes in immune response have also been considered (11, 12). Studies have observed several important differences in immune response between sexes (11). Severe acute respiratory syndrome coronavirus 2 (SARS-CoV-2) enters the cells *via* the cell membrane proteins ACE2 and TMPRSS2. Studies indicate that the expression of both ACE2 and TMPRSS2 is higher in males as estrogen leads to downregulation of ACE2 and androgens upregulate the TMPRSS2 expression (13, 14). The clearance of the virus is also delayed in male relative to female patients as indicated by viral RNA analysis (15, 16). Lower CD4^+^ T proportions and higher monocyte counts have been observed in male COVID-19 patients (11). Iwasaki et al. have demonstrated increased activation of T cells in female patients as compared with males (12). The same study also observed increased IL-8 and IL-18 levels in the plasma as well as induction of non-classical monocytes in male patients (12). These data highlight the differences in immune response of males and females to SARS-CoV-2 infection and suggest that these differences may account for the differential severity of the COVID-19 between sexes.

However, the effect of direct viral sensing as opposed to cytokine exposure to dendritic cells (DCs) and macrophage activation still remains to be determined. One of the problems is that by the time the patient is diagnosed with COVID-19, the innate immune response has progressed too far to be able to determine the initial response of DCs and monocytes against the virus. To overcome this, here we have compared the response to SARS-CoV-2 *in vitro* of cells from the healthy male and female subjects. Gaining a deeper understanding of the interaction between SARS-CoV-2 and the innate immune systems of the hosts may shed light on the development and persistence of inflammation in the lungs. The goal is to use the information to develop or test existing therapeutics, immune modulators that can restore the functions of DCs and monocytes in vulnerable populations. These analyses also provide a potential basis for taking sex-dependent approaches to prognosis, prevention, care, and therapy for patients with COVID-19.

## Materials and Methods

### Blood Donors

Peripheral blood samples were obtained from healthy volunteers (22–57 years old) *via* the help of the Institute for Clinical and Translational Immunology (ICTS), UC Irvine. The ICTS uses the Red Cross criteria for recruiting donors. Individuals with diabetes, infections, medications that modify immune response, and cancer are excluded. The protocol for obtaining the blood is approved by the Institutional Review Board of the University of California (Irvine, CA, USA). Written informed consent was obtained. Cohort description is provided in **Table 1**. Blood was collected in BD Vacutainer tubes with sodium heparin. Fresh peripheral blood mononuclear cells (PBMCs) were used for the experiment.

**Table 1:** Description of the cohort.

Gender	Number of Subjects	Age (years)	Mean ± SD
Male	20	27–57	33 ± 10.7
Female	20	24–57	36 ± 9.2

### Antibodies and Reagents

The following antibody clones were used for staining the cells:

*DCs*: Lineage FITC, HLADR PerCP (clone L-243), CD11c APC (cloneBu15), CD123 BV421 (clone 6H6), CD86 (clone IT2.2), and CD14 BV650 (cloneM5E2) were obtained from BioLegend (San Diego, CA). Live/Dead Fixable Viability Stain 510 was from BD Pharmingen (San Jose, CA, USA).

*CD8T*: CD8 PerCP (clone-SK1), perforin FITC (cloneB-D48), CD107aPE (clone H4A3), and granzyme B AL647 (clone GB11) were obtained from BioLegend.

*Viruses*: Virus-irradiated and heat-inactivated SARS-CoV-2 Isolate USA-WA1/2020 and control irradiated Vero cell lysate were obtained from Bei Resources (NIAID). As per the BEI Resources, gamma irradiation was performed using (5 × 106 RADs) on dry ice, and heat inactivation was performed by heating to 65°C for 30 min. The inactivated viruses are biosafety level 1. The institutional biosafety protocol (IBC) number is 2008-1243.

### Viral Stimulation

PBMCs were isolated from the blood of healthy subjects by density gradient centrifugation. Fresh 2 × 10^6^ PBMCs/2 ml were stimulated with 10 µg of irradiated virus, heat-inactivated SARS-CoV-2 virus, or Vero cell lysate in RPMI 1640 containing 10% fetal bovine serum (Gibco, Thermo Fisher Scientific, USA). After 24 h, half of the cells and supernatants were collected. Cells collected were stained for activation of DCs and monocytes using specific antibodies as described. Supernatants were stored at −70°C for the quantitation of innate cytokines and chemokines. The remaining cells were cultured for another 6 days. Subsequently, the cells were collected and stained for cytotoxic T lymphocytes. Supernatants were stored at −70°C for the quantitation of adaptive immune cytokines.

### Immunophenotyping (Flow Cytometry)

PBMCs collected after stimulation for 24 h were stained with Fixable Viability Stain 510 for live/dead cell exclusion as per the instruction of the manufacturer. The cells were then washed and surface stained for DCs and monocytes using specific antibodies for 30 min at RT in the dark. Subsequently, the cells were washed and fixed using 2% PFA. The required FMO and isotype controls were prepared the same way. Cells were acquired by BD FACS Celesta (Becton-Dickenson, San Jose, CA, USA) equipped with BVR laser. Forward and side scatters and singlets were used to gate and exclude cellular debris. Thirty thousand cells were acquired/sampled. Analysis was performed using FlowJo software (Ashland, OR, USA). DCs were identified by the following phenotypes: lineage^−^, HLA-DR^+^; mDCs-lin^−^/HLA-DR^+^/CD11c^+^; pDC-lin^−^/HLA-DR^+^/CD123^+^; monocytes-CD14^+^/HLA-DR^+^. The expression of CD86 was determined on these gated populations.

For cytotoxic CD8 T-cell staining, the cells collected at day 7 were stained with Fixable Viability Stain 510 for live/dead cell exclusion. After washing, the cells were surface stained with CD8 antibody for 30 min. The cells were then fixed and permeabilized by fix perm buffer (BD Biosciences) and stained with granzyme B and perforin. Appropriate FMO and isotype controls were used. Acquisition and analysis were done as above.

### Multiplex Cytokine/Chemokine Assay

Culture supernatants collected 24 h and 1 week post stimulation were assayed using Multiplex kits (Thermo Fisher Scientific, USA). The specific kit used for the 24-h culture supernatant contained the following analytes: IL-10, IL-6, IL-8, IL-29, CCL-2, TNF-α, IFN-α, CXCL-10, and CCL-19. The kit for the 7-day supernatants contained these analytes: TNF-α, IFN-α, IL-6, IFN-γ, IL-17A, IL-1β, IL-22, and granzyme B. The procedure followed was according to the protocol of the manufacturer. Briefly, the supernatant was mixed with premixed beads (30 cytokines) overnight, and after incubation with detection antibodies and streptavidin-PE for 1 h each, the plate was run on Magpix to identify specific cytokines. IL-18 was detected using specific ELISA kit (Boster Bio, CA, USA).

### Statistical Analyses

Statistical analysis was performed using GraphPad Prism version 9 (GraphPad Inc., San Diego, CA, USA). Paired or unpaired *t*-test was used for comparison between two groups. One-way ANOVA followed by Tukey’s multiple comparison test was used for the analysis of two or more groups. A *p*-value of <0.05 was considered statistically significant. Statistical considerations for each figure are provided in the figure legend.

## Results

### Irradiated SARS-CoV-2 Is a Better Activator of Innate Immune Cells Than Heat-Inactivated SARS-CoV-2

To investigate the early innate immune response to SARS-CoV-2, we activated the PBMCs from healthy subjects with inactivated SARS-CoV-2 and determined the activation of DCs and monocytes. This was done because monocytes, plasmacytoid DCs (pDCs), and myeloid DCs (mDCs) are crucial for antiviral immune responses. pDCs are equipped to detect viruses and are the major type I IFN producers of the immune system in quantities much above the other cells (17). Because of the role of IFN-α in the pathology of COVID-19 (18), it is important to understand the effect of SARS-CoV-2 on pDC activation and functions. Furthermore, mDCs as well as pDCs act as bridges between early innate immune responses and the adaptive immune response in viral infections because of their ability to prime naive T cells, particularly the cytotoxic T-cell and antibody responses required for the elimination of the COVID-19 virus. In addition to DCs, monocytes also prime T-cell responses, but in COVID-19, they have emerged as both the potential source of inflammatory cytokines and the target of the cytokine storm (19–21). The PBMCs were activated with inactivated virus because DCs are resistant to viral replication but can sense various components of viruses including some proteins and viral nucleic acids *via* pathogen recognition receptors such as TLRs and RIG-1, MDA-5, and present antigens to prime T-cell responses (22). We have previously used inactivated influenza virus to activate DCs (23, 24). Monocytes/macrophages do express the ACE2 receptor, but replication of SARS-CoV-1 was also found to be abortive in human macrophages, and inactivated virus was able to induce the secretion of cytokines (25, 26). Another advantage of using the inactivated virus is that these are potential vaccine candidates and knowledge regarding the response of these may help gain insight into vaccine responses.

**Figure 1:**
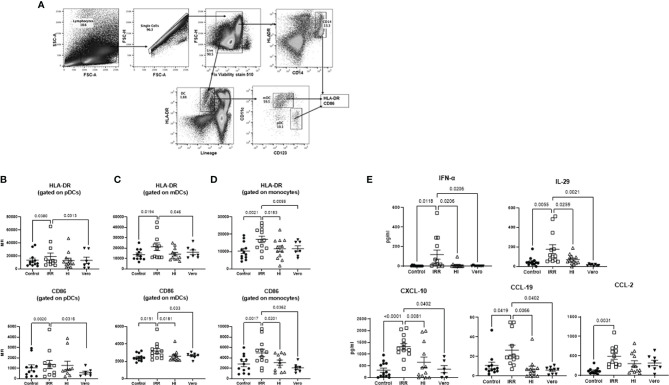
Irradiated severe acute respiratory syndrome coronavirus 2 (SARS-CoV-2) is a better activator of monocytes and DCs than heat inactivated SARS-CoV-2. PBMCs were stimulated o/n with irradiated (IRR) or heat-inactivated (HI) SARS-CoV-2 or Vero cell lysate (Vero). Flow cytometry was used to determine the upregulation of surface markers. Plasmacytoid dendritic cells (pDCs), myeloid dendritic cells (mDCs), and monocytes (mono control) are the unstimulated population gated on pDCs, mDCs, and monocytes. IRR, HI, and Vero are stimulated conditions from the same donors. **(A)** Gating strategy for pDCs, mDCs, and mono (monocytes); MFI of expression of activation markers HLA-DR and CD86 on **(B)** gated pDCs (lineage^−^ HLADR^+^CD123^+^); **(C)** gated mDCs (lineage^−^ HLADR^+^CD11c^+^); **(D)** gated CD14^+^ monocytes using flow cytometry. **(E)** Graphs depict the quantitation of cytokines/chemokines in the supernatant by multiplex. Mean ± SE. *N* = 12 subjects. *p*-value was calculated using one-way ANOVA followed by Tukey’s test.

We compared the response of PBMCs from young (30–42 years of age) subjects to irradiated (IRR) and heat-inactivated (HI) forms of SARS-CoV-2 (USA-WA1/2020) to determine which form is better at inducing an immune response. Initial experiments were done to determine optimal concentration (**Supplementary Figure S1**). Both forms of SARS-CoV-2 worked at 10 µg/ml of protein. Since the virus was grown in Vero cells, the viral formulation may contain Vero cell proteins. Therefore, Vero cell lysate at the same protein concentration (10 µg/ml) was used as a control in initial experiments. PBMCs were stimulated overnight with IRR, HI virus, and Vero cell lysate. Subsequently, the cells were collected and stained for the expression of activation markers, CD86, and HLA-DR. Flow cytometric analysis was performed on gated pDCs, mDCs, and monocytes (**Figure 1A**). The irradiated virus was able to induce a significant increase (*p* < 0.05) in the expression of both HLA-DR and CD86 on pDCs, mDCs, and monocytes as compared with unstimulated cells as well as Vero cell lysate (**Figures 1B**–**D**). In contrast, the heat-inactivated virus was a poor activator of the cells and did not display a significant increase in the expression of these markers over both unstimulated cells and Vero cell lysate (**Figures 1B**–**D**). The difference between the two viral preparations was even more distinct in the case of monocytes where the irradiated virus displayed significantly increased expression of both HLA-DR and CD86 as compared with the heat-inactivated virus (**Figure 1D**). Altogether, these data indicate that the irradiated virus is a better activator of innate immune cells as compared with heat-inactivated SARS-CoV-2.

We also examined the cytokines and chemokines secreted in PBMCs by the IRR and HI virus at 24 h. Supernatants collected were assayed for cytokines using multiplex. Five cytokines/chemokines (eight were tested) showing major differences are depicted in **Figure 1E**. The IRR virus induced significantly increased levels (*p* < 0.05) of IFN-α, IL-29, CXCL-10, CCL-19, and CCL-2 in PBMCs as compared with both unstimulated cells and cells stimulated with Vero cell lysate. IL-6, TNF-α, and CXCL-8 (IL-8) displayed no significant induction by both forms of the virus at the 24-h time point (data not shown). Except for CCL-2, all the other molecules also displayed a significant increase in the IRR virus group compared with the HI virus confirming that the IRR virus is a superior activator of innate immune responses.

### Increased CD8 T-Cytotoxic Responses in PBMCs Stimulated With Irradiated as Compared With Heat-Inactivated SARS-CoV-2

**Figure 2:**
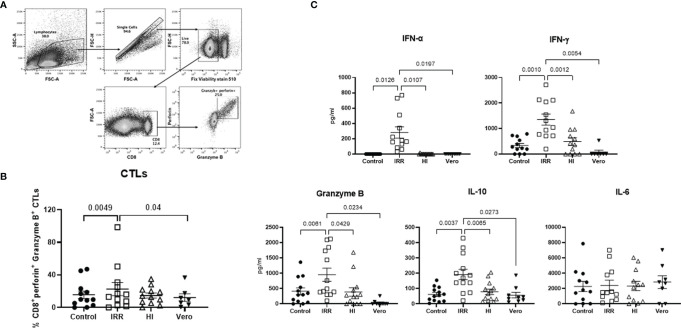
Irradiated SARS-CoV-2 is a better inducer of cytotoxic T lymphocytes than heat-inactivated SARS-CoV-2. PBMCs stimulated with irradiated SARS-CoV-2 (IRR) or heat-inactivated (HI) or Vero cell lysate (Vero) were cultured for 7 days. Cells collected were stained for CD8, perforin, and granzyme B and acquired on a flow cytometer. **(A)** Gating strategy for the identification of these cells. **(B)** Dot plot depicts the % of cytotoxic CD8 T cells. **(C)** Quantitation of cytokines/chemokines in the supernatant by multiplex. Mean ± SE. *N* = 12 subjects. *p*-value was calculated using one-way ANOVA followed by Tukey’s test.

Next, we examined whether the increased activation of early innate immune response by irradiated virus also results in enhanced cytotoxic responses. To investigate this, PBMCs were stimulated for a week with IRR and HI virus. The induction of cytotoxic T lymphocytes (CTLs) was determined by flow cytometry (**Figure 2A**), and the supernatants collected were assayed for soluble cytokines/chemokines with multiplex. The IRR virus induced significantly increased levels of granzyme and perforin expressing CD8 T cells as compared with unstimulated and Vero lysate-stimulated PBMCs (**Figure 2B**). The induction of CTLs by the HI virus was not significant. Examination of the soluble mediators revealed that the IRR virus induced significant increased levels of IFN-α even after a week (**Figure 2C**). The IRR virus also induced significantly increased levels of T-cell cytokines IFN-γ, granzyme B, and IL-10 relative to unstimulated, Vero lysate, and HI virus-stimulated PBMCs (**Figure 2C**). IL-6 displayed no significant induction by IRR or HI virus. Similar results were obtained for TNF-α, IL-17, etc. (data not shown). Overall, IRR induced higher activation of the immune system compared with HI. For the rest of the experiments, only the IRR virus was used.

### Increased Activation of DCs and Monocytes by IRR SARS-CoV-2 in PBMCs From Females Relative to Males

**Figure 3:**
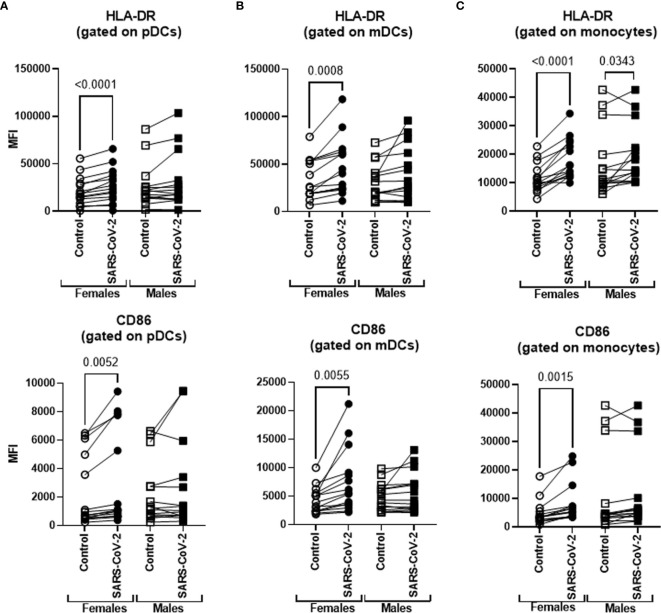
DCs and monocytes from females display enhanced activation in response to SARS-CoV-2 than males. PBMCs were stimulated o/n with irradiated SARS-CoV-2 virus for 24 h. The upregulation of HLA-DR and CD86 was determined using flow cytometry. Lines connect the unstimulated and stimulated conditions from the same subject. **(A)** Gated pDCs (lineage^−^ HLADR^+^CD123^+^); **(B)** gated mDCs (lineage^−^ HLADR^+^CD11c^+^); and **(C)** gated CD14^+^ monocytes. Mean ± SE. Females = 15; males = 15. *p*-value between the unstimulated control and SARS-CoV-2-stimulated condition in males and females was calculated using paired *t*-test (parametric).

SARS-CoV-2 infection causes increased mortality in males than females. Therefore, we examined the differences in activation of DCs and macrophages to IRR virus between males and females. Remarkably, we found increased upregulation of both HLA-DR and CD86 on pDCs (1.3-fold increase for HLA-DR and 1.4-fold for CD86) and mDCs (1.4-fold increase for HLA-DR and 1.7-fold for CD86) after stimulation with SARS-CoV-2 only in females (**Figures 3A, B**). The expression of these molecules in males after stimulation was not significantly different compared with unstimulated controls. The baseline expression of these molecules on pDCs and mDCs was comparable between males and females. In monocytes, the upregulation of HLA-DR was significant in both males (1.2-fold increase) and females (1.6-fold increase) though the significance was higher in females. CD86 was significantly upregulated only in females (1.7-fold increase; **Figure 3C**). There was no significant difference at baseline in both these molecules between females and males. These results indicate that SARS-CoV-2-mediated initial activation of DCs and monocytes is higher in females than males.

### Sex Differences Between Secretion of Soluble Mediators From PBMCs of Males and Females After Stimulation With SARS-CoV-2

**Figure 4:**
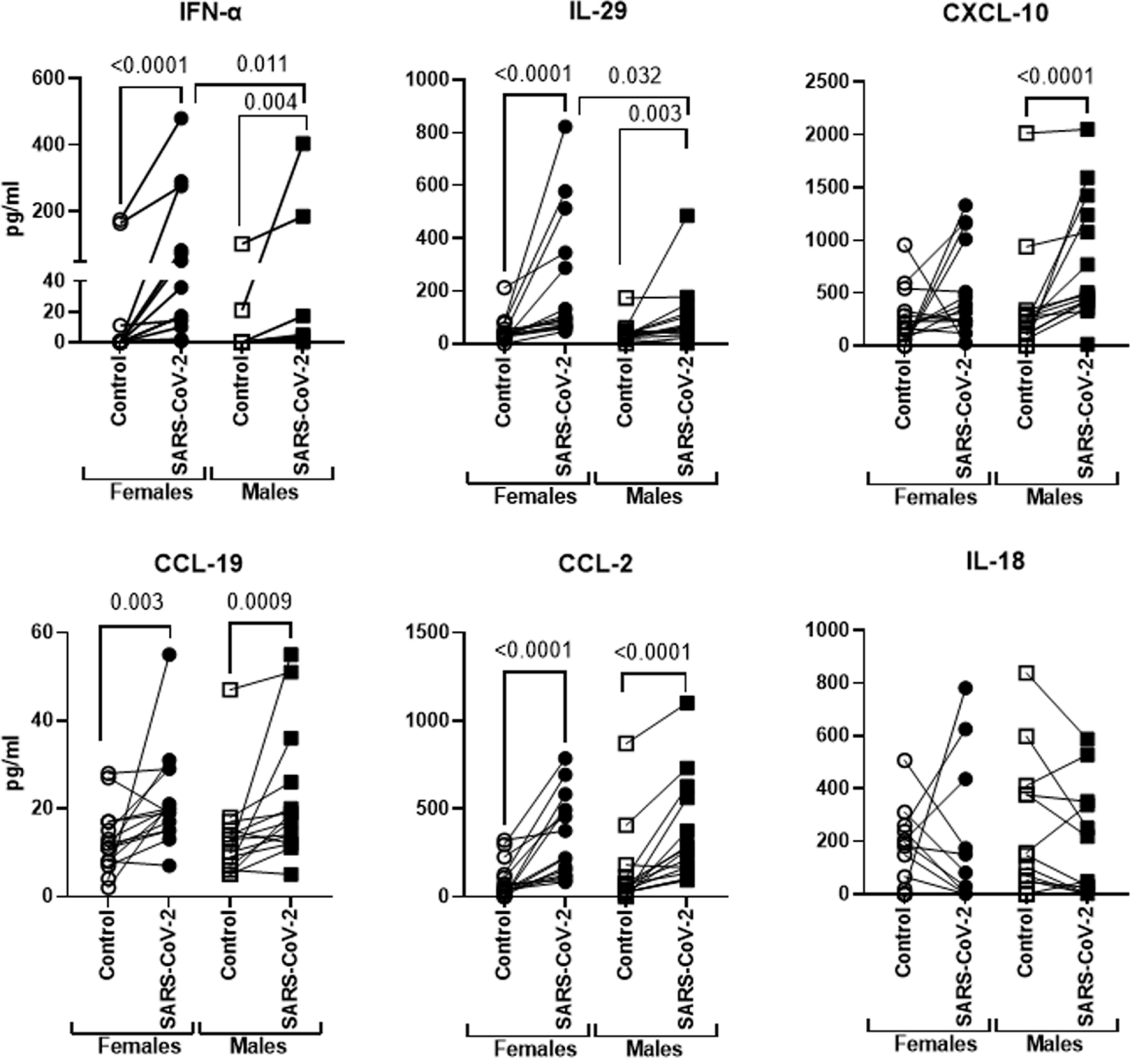
Differential cytokine/chemokine secretion in males and females in response to SARS-CoV-2. PBMCs were stimulated o/n with irradiated SARS-CoV-2 virus for 24 h. Graphs depict the quantitation of cytokines/chemokines in the supernatant by multiplex. Lines connect the unstimulated and stimulated conditions from the same subject. Mean ± SE. Females = 15; males = 15. *p*-value between the control and SARS-CoV-2-stimulated condition in males and females was calculated using Wilcoxon matched pairs signed rank test. Significance between males and females was calculated using unpaired *t*-test (Mann–Whitney test).

Next, we examined the differences in cytokine/chemokine secretion between males and females after overnight SARS-CoV-2 stimulation. We found significantly increased secretion of type 1 and type 3 interferons (IFNs) in PBMCs from females after stimulation with the IRR virus (**Figure 4**). In males, though the virus induced significant secretion of both these molecules, the levels were significantly lower in females. In contrast to innate interferons, the secretion of CXCL-10/IP-10 was significantly higher in males after stimulation with the virus. The levels of CXCL-10 were also increased in stimulated PBMCs from females, but the difference was not significant. CCL-2 and CCL-19 induction in response to the virus was significant in both males and females, and there was no difference between the sexes. IL-18 did not display significant induction in response to the virus and levels before and after stimulation were comparable in both sexes. We had also examined the secretion of IFN-α, IL-29, and CXCL-10 in response to inactivated influenza A virus in an initial few subjects. Stimulation with influenza led to significantly increased secretion of these cytokines in both males and females; however, the difference between both sexes was not significant (**Supplementary Figure S2**). These data therefore indicate that males and females display differences in innate cytokine/chemokine secretion in response to SARS-CoV-2.

### Adaptive Immune Response to SARS-CoV-2 Differs Between Males and Females

**Figure 5:**
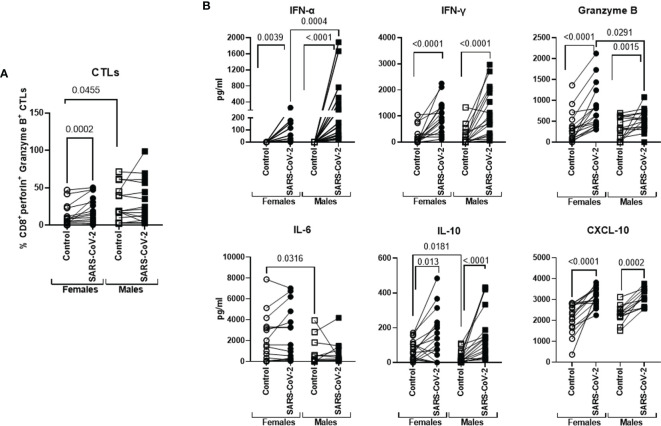
Enhanced induction of cytotoxic CD8 T cells in females than males in response to SARS-CoV-2. PBMCs stimulated with irradiated SARS-CoV-2 were cultured for 7 days. Cells collected were stained for CD8, perforin, and granzyme B. **(A)** Dot plot depicts the % of these cells obtained using flow cytometry. **(B)** Quantitation of cytokines/chemokines in the supernatant by multiplex. Lines connect the unstimulated and stimulated condition from the same subject. Mean ± SE. Females = 15; males = 15. *p*-value between the control and SARS-CoV-2-stimulated condition in males and females was calculated using Wilcoxon matched pairs signed rank test. Significance between males and females was calculated using unpaired *t*-test.

We also investigated the differences in CTL induction and adaptive immune cytokine/chemokine responses between males and females after stimulation with the IRR virus for 1 week. In keeping with increased DC and monocyte activation, we observed significantly enhanced percentages of granzyme B and perforin expressing CD8 T cells in females compared with unstimulated controls. In males, the percentages of the CTLs were significantly higher than those in females at baseline. However, the proportion of CTLs before and after stimulation with the IRR virus was comparable (**Figure 5A**). When we examined the level of T cell and other cytokines and chemokines at this time point, the results were very interesting. In contrast to 24 h, 1 week of stimulation with the virus resulted in significantly increased secretion of IFN-α in males as compared with females (**Figure 5B**). The T-cell cytokines displayed a varied profile between the sexes with granzyme B displaying a significant increase in females compared with males. The induction of IFN-γ and IL-10 after viral stimulation was significant in both males and females, but the levels were comparable in both. However, the levels of IL-10 in females were significantly higher at baseline than those in males. There was no significant induction of IL-6 after stimulation with the virus; however, the level of IL-6 in females at baseline was significantly higher compared with that in males. CXCL-10 levels displayed a significant increase in both males and females after stimulation. In summary, these results indicate an enhanced CTL response to SARS-CoV-2 in females than males. Furthermore, the secretion of type I IFN is delayed in males compared with females.

## Discussion

The COVID-19 pandemic has affected the health of millions of individuals worldwide. There is an urgent need to understand the immune responses underlying the infection to design better therapeutics and prevention strategies. This study focuses on delineating the initial immune response against the virus that has been difficult to investigate in patients due to the gap in timing between when the patient displays disease symptoms and seeks medical attention.

Our results indicate that irradiated SARS-CoV-2 is a superior activator of immune response compared with the heat-inactivated virus. One reason could be that heat inactivation causes major protein denaturation, while irradiation preserves the morphological structure of the virus. Irradiated SARS-CoV-2 was able to activate DCs and monocytes to induce secretion of type I and III interferons. The secretion of other inflammatory cytokines such as TNF-α, IL-1β, and IL-6 was not visible at the early stages of stimulation. On the other hand, we did observe secretion of chemokines, CXCL-10, CCL-19, and CCL-2 by the PBMCs at 24 h. Stimulation with the irradiated virus also induced CTLs as well as T cytokines including IFN-γ, granzyme B, and IL-10. Once again, we did not find induction of TNF-α and IL-6. Previous studies have reported an enrichment in IFN pathways in patients with moderate COVID-19, while patients with severe disease displayed increased inflammatory phenotype combined with decreased IFN production and reduced activation of DCs and monocytes (19, 21, 27–29). Consistent with these data, we find increased IFN signature and decreased inflammatory responses in our healthy young to middle-aged subjects, a population that is not at high risk for COVID-19. Our data also suggest that *in vitro* stimulation with irradiated SARS-CoV-2 can replicate the prominent immune responses observed in COVID-19 patients and, thus, can be used as a model system to test interventions to modulate cytokine and immune responses.

It is well documented that sex influences immune response which in turn changes the outcome of infections (30, 31). This is particularly evident in case of viral infections where higher viral loads are present, and such is the case with hepatitis C virus and with human immunodeficiency virus (HIV), in which males have a larger viral load (32, 33). Several factors account for decreased susceptibility of females to viral infections. These include differences in innate immunity, steroid hormones, and factors related to sex chromosomes. The presence of two copies of X chromosome in females contributes significantly to these differences since the X chromosome controls several immune regulatory genes including TLR7, FOXP3, CXCR3, and CD40L that are helpful in reducing viral load and inflammation (34, 35). To balance the dosage of gene expression due to the presence of 2X chromosomes in females, one X chromosome is randomly inactivated at the early embryo stage. However, some genes escape X chromosome inactivation (XCI) leading to biallelic expression. These include immune genes such as TLR7, CD40L, and CXCR3 (36, 37). The biallelic expression of these escaped genes may provide women with an immune advantage over men and may contribute to the observed differences in COVID-19 between sexes.

Our results indicate that activation of DCs, particularly pDCs, is higher in females. This is attributed to the higher expression of TLR7 in pDCs in females that leads to increased IFN-α production (38). These factors endow females with a more robust ability to control infectious agents. A sex bias has also been reported in COVID-19 with males displaying increased severity as well as higher hospitalization rates (9, 10). Like humans, male mice had a ∼90% mortality rate compared with 20% in females after SARS-CoV infection (39). The underlying mechanisms for this disparity are not well understood. Our study indicates that the immune response of males and females to SARS-CoV-2 is very different. Females mount a robust early innate immune response to SARS-CoV-2 characterized by activation of DCs and monocytes and high type I and type III IFN secretion. This could be due to increased TLR7 signaling since SARS-CoV-2 is a single-stranded RNA virus that can activate the receptor in pDCs to induce IFNs (40, 41). Males, on the other hand, displayed poor innate immune responses with no significant activation of DCs and reduced IFN production at an early time point. The production of IFN-α was, however, higher at day 7 in males indicating a delayed but prolonged secretion. Takahashi et al. have also reported increased IFN-α2 levels in female COVID-19 patients relative to male patients (12). The role of IFN-α in COVID-19 has been hotly contested. Several studies indicate a protective role for the cytokine, while others deem it to be responsible for long-term disease consequences, such as fibrotic findings in SARS patients (18, 39, 42–46). Further studies suggest that the timing and level of IFN-α secretion dictates the difference in it being protective or harmful. For example, early IFN responses were higher and waned over time in patients with moderate disease. In contrast, IFN secretion was lower to start with but displayed an increase throughout infection in patients with severe cases (27). Likewise, administration of IFN in mice 1 day after SARS-CoV infection was protective, while later administration led to increased inflammation and mortality (44). The success of prophylactic IFN in protecting from COVID-19 infection supports its early beneficial effects. In 3,000 medical staff administered intranasal IFN-α as a prophylactic during a COVID-19 outbreak, no new infections were seen during a 28-day observation period compared with 100 new infections in untreated staff at neighboring Wuhan hospitals (47). Prophylactic IFN-α has also been confirmed in Syrian hamster models to restrict COVID-19 disease progression but showed little effect after the onset of symptoms (48, 49). The sex differences regarding late IFN levels are unknown. Our study indicates that in response to SARS-CoV-2, robust activation of DCs and monocytes along with early IFN secretion in females may be beneficial, while the decreased activation coupled with delayed but prolonged IFN secretion in males may be detrimental. These differences may contribute to the increased mortality in males due to COVID-19.

Another interesting observation from our study was the secretion of high levels of CXCL-10 in males as compared with females at an early time point. Takahashi et al. also observed elevated levels of CXCL-10 in male COVID-19 patients compared with male controls, while the levels were lower in female COVID-19 patients relative to female controls (12). IFN-γ-inducible protein 10 (IP-10 or CXCL-10) is known to be a major chemokine involved in antiviral responses in the respiratory tract (50). It acts as a chemoattractant for monocytes/macrophages, DCs, NK cells, and T cells. In the lungs, it is involved in the recruitment of CXCR3-positive macrophages that produce high levels of IL-6 (51). Elevated levels of CXCL-10 have been reported in both the plasma and bronchial alveolar lavage fluid (BALF) and were associated with disease severity in viral infections (50). Similar findings have been reported for COVID-19 (51–53). CXCL-10 was consistently found to be elevated in the serum of patients with severe COVID-19. A positive correlation was also observed with increased disease severity and increased risk of mortality (51, 54). Bioinformatics analysis of GEO datasets for COVID-19 patients also identified CXCL-10 as the key cytokine linked to cytokine storm (55). Increased CXCL-10 production early in the response by males may thus be another contributing factor for the increased progression to severe COVID-19.

Takahashi et al. (12) had observed increased IL-8 in IL-18 levels in the plasma of male patients infected with SARS-CoV-2 compared with females. Stimulation with SARS-CoV-2 does not induce IL-8 or IL-18 at 24 h in our study. This suggests that the source of IL-8 and IL-18 observed in the study of Takahashi et al. is most likely not the immune cells present in PBMCs of healthy donors. The differences between *in vitro* and *in vivo* that include cell culture medium and viral load can also be responsible for the discrepancy.

Consistent with enhanced innate immune responses, females also displayed increased CTL activity in response to SARS-CoV-2. It is known that females in general exhibit increased CD8 responses (56). This is attributed to the presence of estrogen response elements in the CD8 promoters. In addition, the increased activation of TLR7 also enhances CD8 T-cell activation in females compared with males (11, 57). The increased CTL activity in females may be able to control the viral load as has been reported in SARS-CoV (58). Increased T-cell activation in females relative to males in COVID-19 has also been reported (12). They also found that lower T-cell responses were associated with more severe disease in male patients.

Our study has several limitations. For our studies, we have used total PBMCs that contain a mix of different cell populations. Though this is more physiological as these cells are also present together in the body, the limitation is that the possible interactions between the different cell types upon stimulation may have affected the results and observations. We cannot specify the cell type producing the cytokines. Furthermore, we have used medium containing FBS for our study. FBS contains low levels of hormones that can influence the response of cells. Media components like phenol red can also activate estrogen receptors (59). Though we do not find differences in the response between males and females to influenza A using the same media, nevertheless, the media components may have contributed to differences in sex response observed here. It would have also been beneficial to include another control virus such as a DNA virus besides influenza to determine if the difference in immune response between males and females observed in our study is SARS-CoV-2 specific or extends to other viruses. Another limitation is that this study is focused only on certain innate and adaptive immune responses and does not consider the changes in functions of all the immune cells. In addition, sex differences in SARS-CoV-2 are influenced by multiple factors including differences in genetics, behavior, and hormones between sexes. An example is smoking that is more common in males, and smoking has been shown to enhance the expression of ACE2 expression in the lung (60). The present study is limited to differences in immune responses between sexes.

In summary, our data indicate that *in vitro* stimulation with irradiated SARS-CoV-2 simulates the early innate immune responses. In addition, we find that females display significant activation of innate immune cells including pDCs, mDCs, and monocytes. This coupled with early IFN secretion leads to robust induction of CTLs. The increased immune response against SARS-CoV-2 in females may be beneficial. In contrast, the activation of innate immune cells as well as induction of CTLs is not significant in males. The slower secretion of IFN-α along with increased secretion of CXCL-10 may be responsible for worse COVID-19 outcomes in males.

## Data Availability Statement

The raw data supporting the conclusions of this article will be made available by the authors, without undue reservation.

## Ethics Statement

The studies involving human participants were reviewed and approved by UC Irvine IRB. The patients/participants provided their written informed consent to participate in this study.

## Author Contributions

SA performed the experiments and analysis. JS assisted in some flow experiments. TT helped in analysis and discussion. AA wrote the manuscript and supervised the experiments. All authors helped in the discussing and editing of manuscript. All authors contributed to the article and approved the submitted version.

## Funding

The study was supported by grants R00RG2352 and R01RG3735 to AA from the University of California Office of the President (UCOP).

## Conflict of Interest

The authors declare that the research was conducted in the absence of any commercial or financial relationships that could be construed as a potential conflict of interest.

## Publisher’s Note

All claims expressed in this article are solely those of the authors and do not necessarily represent those of their affiliated organizations, or those of the publisher, the editors and the reviewers. Any product that may be evaluated in this article, or claim that may be made by its manufacturer, is not guaranteed or endorsed by the publisher.
