# Supplementary material for: Sex-Related Differences in Innate and Adaptive Immune Responses to SARS-CoV-2
Source: Front Immunol. 2021 Oct 20;12:739757. doi: 10.3389/fimmu.2021.739757 (PMC8563790; doi:10.3389/fimmu.2021.739757)
Supplement: Supplementary file 1 [file DataSheet_1.pdf]

Supplementary figure 1

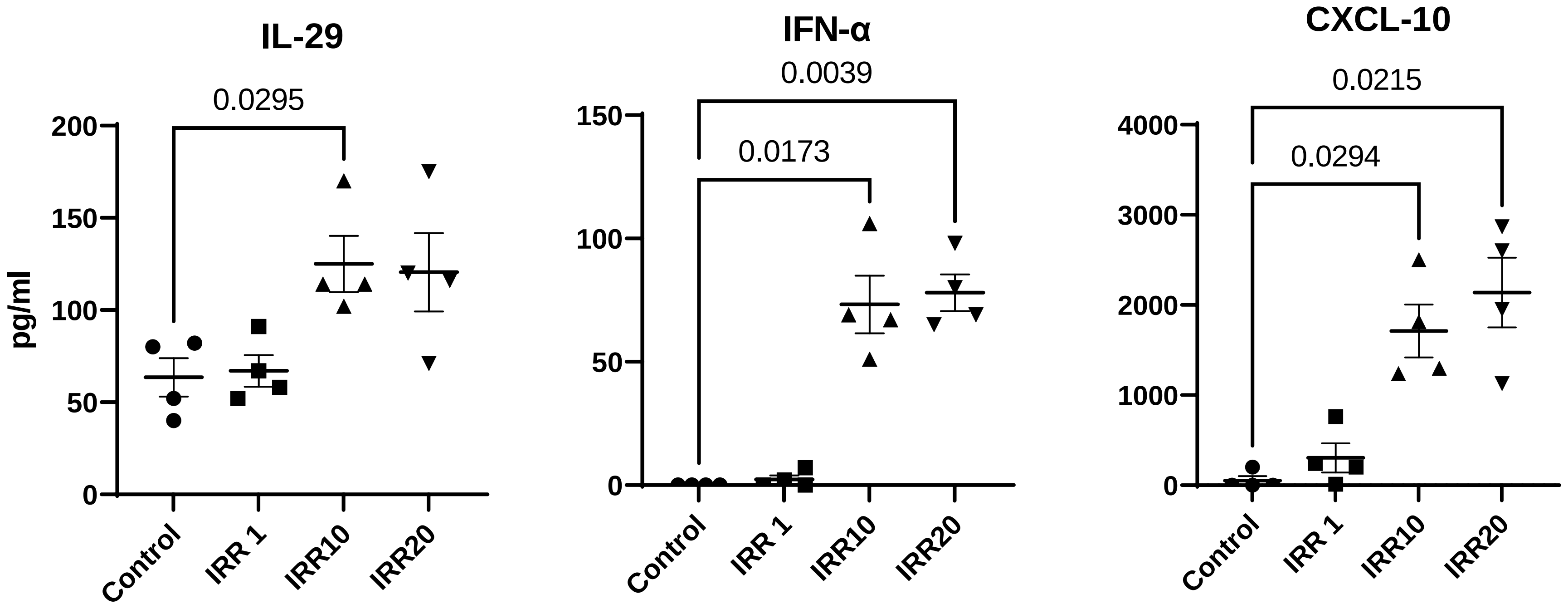

PBMCs were stimulated o/n with irradiated SARS-CoV-2 at concentrations ranging from 1µg/ml-20µg/ml. Graphs depict the quantitation of cytokines/chemokines in the supernatant by specific ELISAs (IL-29 and IFN-α from Biolegend and CXCL-10 from BD Biosciences). Mean ± S.E. N=4 subjects. P value was calculated using one way ANOVA.

Supplementary figure 2

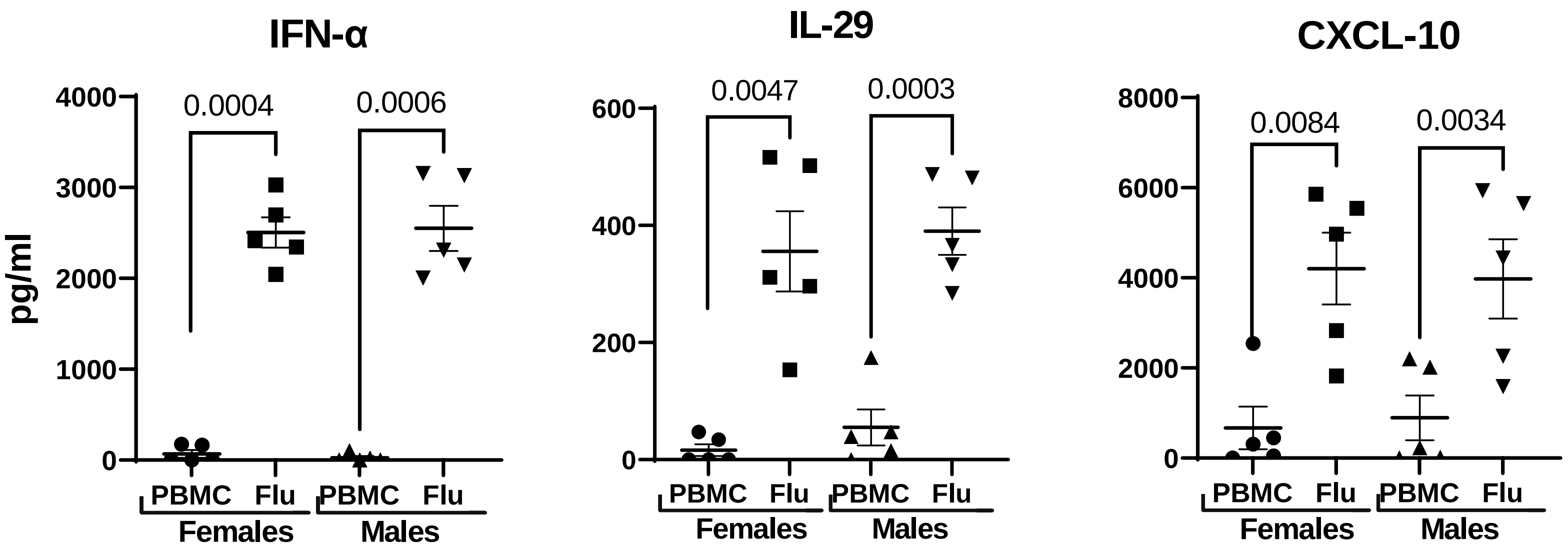

PBMCs were stimulated o/n with inactivated influenza virus A at 1μg/ml. Graphs depict the quantitation of cytokines/chemokines in the supernatant by multiplex. Mean $\pm$ S.E. N=5 subjects. P value was calculated using paired t test.

IFN-α 24h(pg/ml)

Female		Male	
PBMC	IRR	PBMC	IRR
11.1	14.3	0	3.5
0	0.8	0	0
0	17.3	0	0
0	290.9	0	17.3
0	36	21	404
165	277	103	186
176	480	0.4	0.5
0	74	0	0
0	10	0	4
0	52	0	0
0	17	0	0
0	2	0	0
0	84	0	2
0	1	0	4
1	2	0	5

IL-29 24h(pg/ml)

Female		Male	
PBMC	IRR	PBMC	IRR
213	345	39	115
42	105	30	60
26	59	39	54
36	66	61	485
38	514	15	0
0	47	0	23
78	578	174	177
38	96	47	42
84	823	28	65
23	132	22	32
40	93	50	36
16	288	24	54
30	62	31	150
32	72	38	103
55	80	24	73

CXCL-10 24h(pg/ml)

Female		Male	
PBMC	IRR	PBMC	IRR
153	467	232	1426
63	367	118	1240
595	1173	940	1079
0	1011	0	1594
958	26	220	420
0	1161	0	16
545	512	2015	2053
0	1332	219	770
106	205	115	419
179	395	276	458
166	119	304	507
288	234	341	485
230	234	66	357
221	322	105	443
328	238	279	330

PBMC represents the unstimulated condition and IRR the PBMCs stimulatedcondition with irradiated SARS-CoV2
Values for unstimulated and stimulated are paired and from the same donor

HLADR (pDC) MFI

Female		Male	
pDC	IRR	pDC	IRR
34300	42053	36957	65445
4621	6942	1812	1468
521	688	13873	11995
17657	32144	22087	18052
8491	12922	15639	12601
5389	6103	7563	11668
13043	15023	22032	28154
20968	27231	13251	18184
18779	23639	25836	32552
15487	20579	24877	25326
27512	37959	20733	22527
29376	33915	17267	16852
14500	19350	14569	12722
55608	65678	86347	103562
43666	52028	69265	76963

HLADR (mDC) MFI

Female		Male	
mDC	IRR	mDC	IRR
26235	28567	19466	22061
18843	22927	10083	11268
6767	11265	9827	10168
25236	40047	10134	9656
18867	44982	11767	11282
12241	19579	20887	15405
53143	60007	37428	44099
38681	61144	56928	76658
78794	118285	31644	32173
54174	63059	35542	95957
54130	65894	40409	48852
50493	88910	72585	83574
18835	24995	18075	26607
19262	23928	19859	24752
18743	25181	57720	61628

HLADR (monocytes) MFI

Female		Male	
CD14	IRR	CD14	IRR
19395	26572	7390	22348
4358	12449	6140	13419
6756	16282	8259	10176
9647	11967	42700	36825
11968	21135	33934	33690
17814	22760	37240	42686
11034	24883	19885	21479
22816	34349	14700	14445
7516	12916	9295	19448
14384	24353	10950	10574
11597	13961	15037	18135
10259	10011	10239	10140
9822	16104	9415	13962
8966	13794	9688	10578
8418	12928	11256	13620

CD86 (pDC) MFI

Female		Male	
pDC	IRR	pDC	IRR
700	1090	1109	1056
1111	1518	247	310
237	379	1203	1365
652	835	2739	2694
426	597	2774	3410
6495	7753	5878	9469
6126	7877	1689	1404
737	1005	533	727
495	689	674	757
502	1027	841	961
975	1138	759	721
559	644	644	545
6331	9430	1329	658
4988	8046	6436	9449
3582	5271	6627	5950
2261.0667	3153.26667	2232.133	2631.73333

CD86 (mDC) MFI

Female		Male	
mDC	IRR	mDC	IRR
3224	3427	2554	2962
5097	7706	2280	2624
2039	2400	9827	10168
2406	4014	2181	2141
2416	2974	2224	2369
2308	3703	3371	2703
1854	2192	4297	4311
5091	16075	6279	7204
7292	9149	3085	3129
2947	5391	6942	7152
3916	5570	8787	11232
10011	21217	5316	5757
5156	6181	3844	3634
5523	8668	6168	6960
6284	14068	5796	13139
4370.933	7515.666667	4863.4	5699
1.719465			

#DIV/0!

CD86 (monocytes) MFI

Female		Male	
CD14	IRR	CD14	IRR
3626	5437	853	2045
890	4201	998	5099
1949	3567	8259	10176
1946	3451	42700	36825
17814	22760	33934	33690
11034	24883	37240	42686
3322	6603	3158	3730
2508	3546	4342	4830
4445	6549	2221	2139
6676	14625	4345	6168
2691	3692	3542	3802
3111	7489	4713	5818
3608	5379	3495	4984
3480	5147	3920	6667
5472	7351	4699	6712
4838.133	8312	10561.267	11691.4
1.718018			

pDC, mDC and CD14 represents the unstimulated condition and IRR the stimulated with irradiated SARS-CoV2
Values for unstimulated and stimulated are paired and from the same donor

GranzymeB Perforin + CTLs (% cells)

Female		Male	
CD8T	IRR	CD8T	IRR
0.1	0.49	18.7	24.7
5.26	7.51	2.64	2.62
9.75	15.9	40.1	35.1
23.2	30.8	45.1	98.7
8.88	10	71.6	69.4
2.64	2.62	60.7	61.1
0.44	0.72	16.9	16.9
0.15	1.57	9.95	13
47.1	50.4	39.3	44.3
8	14	3.2	3.18
4.21	36.1	2.49	7.05
41.8	47.4	18.1	9.95
11.7	18.9	2.77	3.54
25.1	48.3	62.1	56.5
3.28	2.56	3.21	2.66
2.4	26.4	18.9	21.7
4.77	4.92	17.9	3.82

Granzyme B Day 7 (pg/ml)

Female		Male	
PBMC	IRR	PBMC	IRR
0	408	0	0
541	1437	0	761
914	1732	0	0
46	890	0	1077
0	1242	0	541
1362	2123	104	207
349	491	140	384
83	388	292	391
48	304	330	347
263	861	298	301
387	851	629	711
96	320	640	548
213	429	692	801
715	809	601	696
123	637	333	500
274	640	477	606
328	450	592	521

IFN-α Day7(pg/ml)

Female		Male	
PBMC	IRR	PBMC	IRR
0	179	0	1665
0	0	0	1888
0	0	0	162
0	158	0	768
0	0	0	510
0	0	0	129
0	0	0	62
0	0	0	223
0	0	0	84
0	0	1	28
0	30	1	23
1	261	1	25
1	11	1	35
1	10	1	368
1	58	1	212
1	4	1	53
1	117	1	129

PBMC represents the unstimulated condition and IRR the PBMCs stimulated with irradiated SARS-CoV2
Values for unstimulated and stimulated are paired and from the same donor
